# Microbial Sampling Using Interdental Brushes and Paper Points around Teeth and Implants: A Pilot Study for Comparison

**DOI:** 10.3390/diagnostics13061054

**Published:** 2023-03-10

**Authors:** Tobias M. Janson, Yann Gager, Christian R. Hatz, Anne-Katrin Köhler, Stefanie J. Gartenmann, Patrick R. Schmidlin

**Affiliations:** 1 Clinic of Conservative & Preventive Dentistry, Division of Periodontology & Peri-Implant Diseases, Center of Dental Medicine, University of Zurich, 8032 Zurich, Switzerland; 2 ParoX Dental Gmbh, 04103 Leipzig, Germany; 3 Dr. Stefanie Gartenmann Dental Practice, 6330 Cham, Switzerland

**Keywords:** periodontitis, peri-implantitis, microbial sampling, interdental cleaning, molecular genetics

## Abstract

Inflammatory periodontal and peri-implant diseases follow dysbiotic shifts in a susceptible host. A well-established tool for microbial sample collection is the use of paper points. The purpose of this pilot study was to evaluate the use of interdental brushes compared to paper points. Biofilm samples were collected with paper points and later interdental brushes from ten patients. Five patients were represented with a community periodontal index of treatment needs (CPITN) of 0–2 around the teeth and an implant with PPD ≤ 5 mm and no radiological bone loss. The remaining five patients had a CPITN ≥ 3 and one implant with peri-implantitis. Microbial samples were analyzed with quantitative polymerase chain reaction (qPCR) and next-generation sequencing (NGS). The results showed higher amounts of DNA in samples taken by interdental brushes but also higher Ct values. Both methods detected *Filifactor alocis*, *Porphyromonas gingivalis*, *Prevotella intermedia*, *Tannerella forsythia,* and *Treponema denticola* in the majority of samples, while *Aggregatibacter actinomycetemcomitans* was rarely found. A microbial dysbiosis index showed comparable or higher values in sites with no periodontitis/peri-implantitis with interdental brushes. The results of this pilot study indicate that interdental brushes might be a valid technique for microbial sampling and particularly advantageous in the early detection of dysbiotic shifts around teeth and implants. Larger studies with more participants are needed to validate the proposed microbial sampling method with interdental brushes.

## 1. Introduction

Inflammatory periodontal and peri-implant diseases and conditions represent mostly chronic forms, which attack—if untreated—the surrounding soft and mineralized supporting tissues. The main related entities, i.e., gingivitis/mucositis and periodontitis/peri-implantitis, respectively, share similarities but also display differences, for example, in terms of the microbiome or rate of progression [1]. However, oral bacteria play a pivotal role in the initiation and progression of both but remain influenced by several shared modifying factors, such as the susceptibility of the individual patient [2,3].

While studies [4,5,6] have shown that the bacterial load of infected teeth or implants may be significantly higher than that of healthy ones, both nonshedding structures also tend to harbor more pathogenic species, mainly from the so-called orange and red complex [4,5,7,8]. In particular, bacteria of the latter (i.e., *Porphyromonas gingivalis*, *Tannerella forsythia*, and *Treponema denticola*) and notably also *Aggregatibacter actinomycetemcomitans* have been identified and associated with accentuated degradation patterns of the soft and hard tissue around the teeth and implants since these bacteria produce proteolytic enzymes and other virulence factors, which trigger an immune response of the host [5,9,10].

To determine the composition of the biofilm, various sampling techniques have been developed. Sampling usually encompasses plaque harvesting from subgingival areas using sterile paper points, which are inserted in preselected sites. In previous studies, the comparability of oral biofilm samplings with curettes and sterile paper points was investigated and found to be mostly comparable [11]. Another option for microbiological sample collection, which may require less pre-evaluation and may be more comfortable for patients, might be found in the use of interdental brushes. The interdental space is less keratinized, and bacterial products such as endotoxins, peptides, and organic acids produced by the biofilm can easily migrate through the tissue and trigger defense reactions. Unlike on lingual or buccal smooth surfaces, there is no self-cleaning mechanism in the interdental space. Thus, the biofilm can continue to develop there only slightly disturbed by simple tooth brushing or the natural flow of saliva and may offer an ideal sampling site [12].

In conventional microbial sampling with paper points, the pocket depth must usually be measured in advance to perform microbial probing. Accordingly, a paper point is inserted at the deepest sites of the four quadrants for at least ten seconds to obtain the most representative result [13]. This complicates the clinical procedure organizationally and especially for healthy patients or patients in a transitional phase where actual pockets are not yet present technically [14]. Although microbial examinations are not currently recommended as a routine diagnostic tool in cases of biofilm-induced periodontal or peri-implant disease, new findings are desirable, especially in the area of early detection [15,16,17]. Technically, the placement and collection of specimens with paper points are difficult. On the other hand, cleaning the interdental spaces and the associated removal of plaque with interdental brushes is a routine procedure. Papaioannou et al. [18] assessed the role of interdental brushing application in the translocation of periodontopathogens. For this purpose, interdental brushes were first used in patients with uncontrolled periodontitis, and then the same sites were tested with paper points. The study found that anaerobic cultivation could detect most of the same periodontopathogens, and thus interdental brushing is a possible route for the translocation of periodontopathogens. If a minimally invasive technique with standardized interdental brushes as part of oral hygiene instruction led to results comparable to subgingival sampling with paper points, patients and dentists would benefit from an “instant” test, which reduces the number of treatment appointments and improves patient comfort.

This study aimed to compare two sampling methods as a pilot study—interdental brushes and paper points—for the microbial diagnosis of teeth and implants as well as to identify the most appropriate method. We hypothesize that interdental brushes are suitable for collecting bacterial DNA for intraorally microbial testing. Therefore, it might be possible to collect clinically relevant amounts of periodontopathogenic bacterial DNA in the area around the teeth and dental implants with interdental brushes.

## 2. Materials and Methods

This study was designed as a prospective, two-arm controlled pilot study and evaluated by the ethical committee of the canton of Zurich, Switzerland (KEK) (BASEC-Nr. Req-2020-00205). This entire study was carried out in accordance with the Helsinki protocol.

### 2.1. Study Population and Periodontal/Peri-Implant Status Evaluation

Patients were recruited from the pool of the involved clinic. Subjects enrolled in this study had to fulfill the following criteria: (1) aged ≥ 18 years; (2) systemically healthy; (3) no intake of systemic antibiotics and no periodontal surgery in the 6 months preceding the study; and (4) smoking status: ≤10 cigarettes/day.

One to four weeks prior to specimen collection, the community periodontal index of treatment needs (CPITN) was recorded using a PCP 12 periodontal probe (Hu-Friedy Mfg. Co., LLC., 60,528 Frankfurt a. M., Germany). Scores 0 to 4 were defined as described by Ainamo et al. [19]:

– Score 0: Pocket probing depth ≤ 3.5 mm, no bleeding, and no calculus;
– Score 1: Pocket probing depth ≤ 3.5 mm, bleeding, and no calculus;
– Score 2: Pocket probing depth ≤ 3.5 mm, bleeding, and calculus;
– Score 3: Pocket probing depth 4–5.5 mm, ±bleeding, ±calculus;
– Score 4: Pocket probing depth ≥ 6 mm, ±bleeding, ±calculus.

In total, 10 patients were recruited for the study. Therefrom, 5 patients presented healthy patients without either active periodontitis (non-P) or peri-implantitis (non-PI) with a CPITN score of 0–2, and 5 patients presented with periodontitis (P) and peri-implantitis (PI), i.e., a CPITN of 3 or 4. Non-PI was defined as pocket probing depth (PPD) ≤ 5 mm, bleeding on probing (BoP) +/−, and no radiological bone loss. PI was characterized by PPD ≥ 6 mm, BOP +, and radiological bone loss >2 mm [20].

### 2.2. Sampling Method

**Figure 1:**
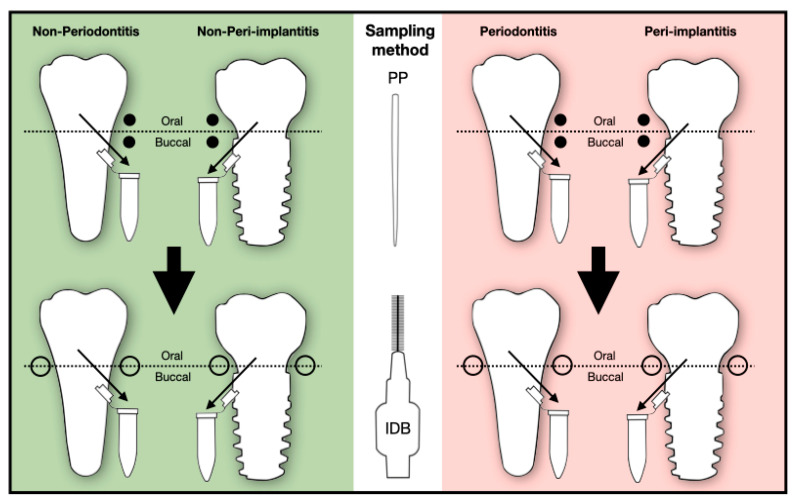
Flowchart of the sampling method. Ten patients were included in the study. While five patients belonged to the non-periodontitis/non-peri-implantitis group, the remaining five patients were part of the periodontitis/peri-implantitis group. First, biofilm samples were taken buccally and orally with paper points (PP). Then, specimens were collected mesially and distally with interdental brushes (IDB). The tips of the paper points and interdental brushes were transferred to separate empty collecting tubes.

Prior to microbiological sampling, the determined sites were first isolated with cotton rolls to avoid salivary contamination. After careful removal of supragingival plaque, subgingival plaque samples were taken using sterile paper points (iso 60); two paper points per site (buccally and orally) were left in place for 15 s as described in previous studies [11,18,21]. During the complete procedure, great care was taken not to provoke any sulcular bleeding. Immediately afterward, interdental brushes derived from an unopened pack (0.5 mm in diameter, produced by Tepe, Malmö, Sweden) were inserted in the interdental space mesially and distally to collect a second microbiological sample. The sampling method is illustrated in Figure 1.

The tips of the paper points and interdental brushes were cut off with sterile scissors and transferred to separate empty collecting tubes which were sent to IAI AG (Institut für Angewandte Immunologie IAI AG, Zuchwil, Switzerland) in their conventional boxes.

### 2.3. Molecular Genetics

All samples were evaluated by IAI AG. The DNA concentration (ng/µL) was obtained using the Qubit Fluorometric Quantitation (Thermo Fischer Scientific, Waltham, USA). In addition, two molecular analyses were performed: iai PadoTest^®^ (IAI AG, Zuchwil, Switzerland) based on quantitative PCR (qPCR) and PadoBiom^®^ (IAI AG, Zuchwil, Switzerland) based on next-generation sequencing (NGS).

qPCR

iai PadoTest^®^ is based on the method of quantitative PCR to quantify human DNA, bacterial DNA, and six bacterial species strongly associated with periodontal disease: *Aggregatibacter actinomycetemcomitans* (*A. actinomycetemcomitans*), *Filifactor alocis* (*F. alocis*), *Porphyromonas gingivalis* (*P. gingivalis*), *Prevotella intermedia* (*P. intermedia*), *Tannerella forsythia* (*T. forsythia*), and *Treponema denticola* (*T. denticola*). Based on quantitative results, PadoTest types ranging from 1 to 5 (low to high degrees of disease severity and corresponding therapy recommendations) were also calculated. Delta-Ct was calculated as the difference in the Ct values between human and bacterial DNA.

NGS

PadoBiom^®^ is based on the method of next-generation sequencing, namely, a customized probe panel to sequence a selection of bacterial species found in oral microbiomes. Based on this probe panel, a level of imbalance in the microbiome—the microbial dysbiosis index (MDI)—is obtained. The MDI is calculated as the ratio of the coverage of a selection of bacterial species associated with health and the coverage of a selection of bacterial species associated with periodontitis. Higher values of MDI are associated with higher levels of dysbiosis. Statistical comparative analyses are used to identify patients who, due to above-average deviating microbiome structures, are expected to have increased disease progression and would benefit from intensified therapy measures [22].

### 2.4. Data Analysis

The data were analyzed, and the graphics were produced using the software R [23] with the package ggplot2 [24].

## 3. Results

### 3.1. DNA Concentration

**Figure 2:**
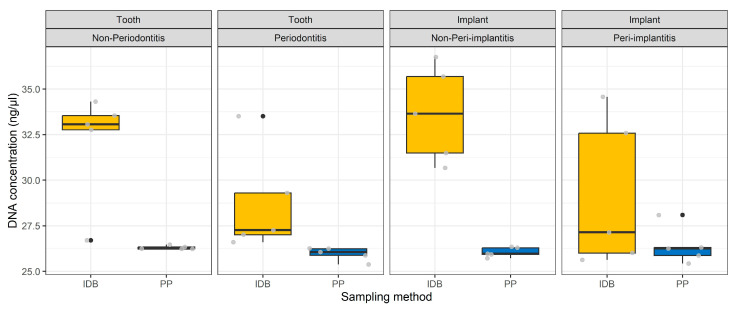
Boxplots of DNA concentration (ng/µL) in relation to sampling site, status, and method. IDB stands for interdental brushes, and PP stands for paper points.

The DNA concentration (ng/µL) was generally higher in patients sampled with interdental brushes but also more variable than in patients sampled with paper points (Figure 2). While the DNA concentration of samples taken with PPs was very similar between the groups, higher DNA concentrations were obtained with IDB around teeth without periodontitis or peri-implantitis compared to periodontitis and peri-implantitis.

### 3.2. qPCR

**Figure 3:**
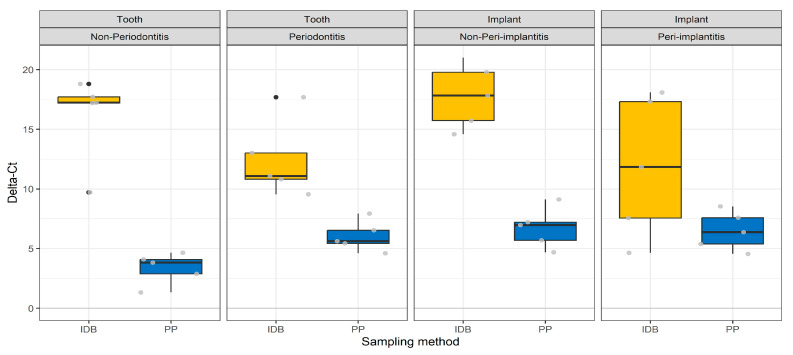
Boxplots of Delta-Ct in relation to sampling site, status, and method. IDB stands for interdental brushes, and PP stands for paper points.

Delta-Ct (ng/µL) was systematically higher and more variable for patients sampled with IDB in comparison to patients sampled with PP (Figure 3).

**Figure 4:**
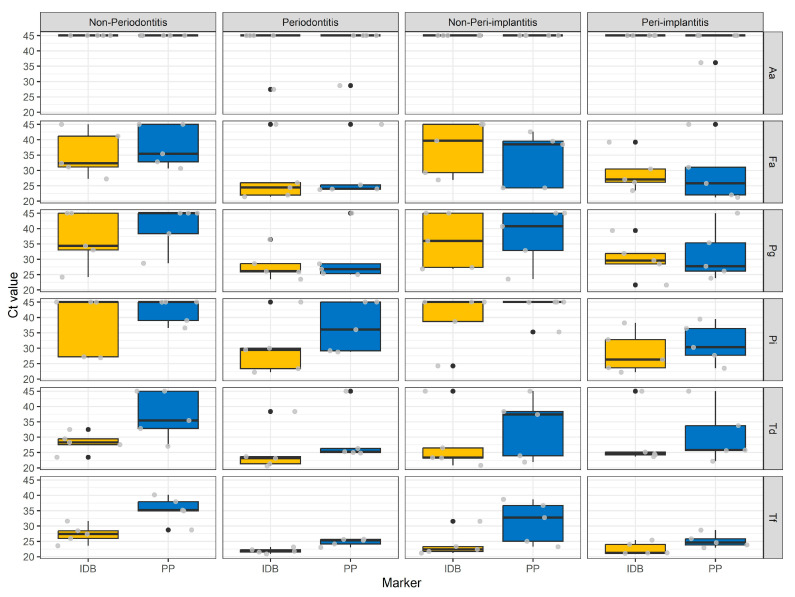
Boxplots of Ct values in relation to sampling status and method for the six different marker bacteria of iai PadoTest^®^: Aa for Aggregatibacter actinomycetemcomitans; Fa for *Filifactor alocis*; Pg for *Porphyromonas gingivalis*; Pi for *Prevotella intermedia*; Td for *Treponema denticola*; and Tf for *Tannerella forsythia*. A Ct value of 45 means absence of the species. IDB stands for interdental brushes, and PP stands for paper points.

*A. actinomycetemcomitans* was absent from the non-P/PI samples (Ct values set a value of 45; Figure 4) while being rarely detected in the P and PI groups. The five other marker bacteria of periodontitis were detected in the majority of samples. Higher concentrations (lower Ct values for all medians and the majority of boxplots) were detected for *T. denticola* and *T. forsythia* for patients sampled with IDB in comparison to patients sampled with PP. A strong overlap of the Ct values for *F. alocis* and *P. gingivalis* was observed for patients both sampled with IDB and PP. For *P. intermedia*, all boxplots were overlapping, but the median concentrations in the P and PI groups were higher (lower Ct values) with IDB.

### 3.3. NGS

**Figure 5:**
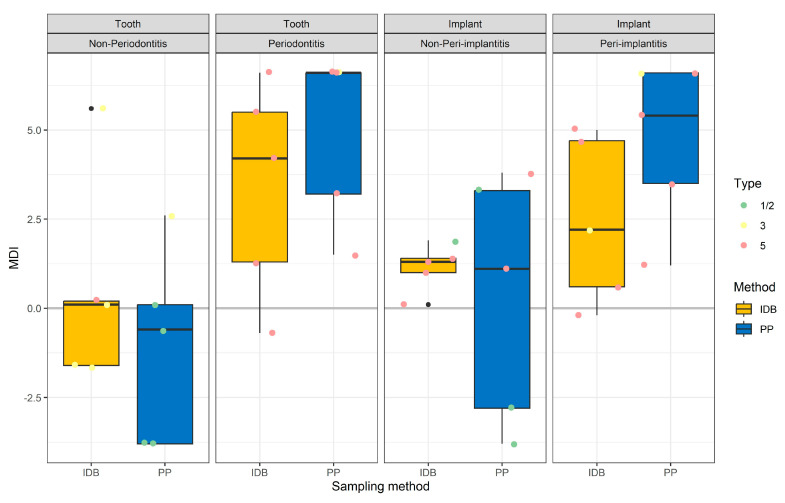
Boxplots of MDI (PadoBiom^®^) and points of type (iai PadoTest^®^) in relation to sampling site, status, and method. IDB stands for interdental brushes, and PP stands for paper points.

All sampling methods and sites showed an overlap in the MDI values and PadoTest types (Figure 5). For non-P and non-PI sites (healthy sites for teeth and implants), the lowest MDI values were observed as well as a higher number of PadoTest Type 1/2 with patients sampled with PP in comparison to patients sampled with IDB. On the contrary, the highest MDI values were observed for P and PI sites (diseased sites for teeth and implants) sampled with PP in comparison to IDB.

## 4. Discussion

This pilot study investigated the feasibility of microbiological testing with commercially available interdental brushes and compared it to common sampling with paper points. To the authors’ best knowledge, no other study ever compared these two microbiological sampling techniques so far.

The results showed no differences in most of the outcome parameters comparing the two sampling methods within the used commercially available test kits (iai PadoTest^®^ and Padobiom^®^, IAI AG, Zuchwil, Switzerland). First of all, total DNA concentration was higher with the test method (IDB) as compared to the control (PP), especially in healthy or inflamed (gingivitis/peri-implant mucositis) sites. Nevertheless, the Delta-Ct was also higher with the test method. The higher values for DNA concentration and Delta-Ct when sampled with IDB can be explained by the larger contact area of IDB with the marginal tissues and the more invasive sampling under the in and out motion of the brush. Therefore, more human DNA from epithelial cells of the marginal gingiva was probably retained on the bristles. This again lowered the ratio of bacterial DNA in the sampling mass. Consistent with the literature, higher bacterial loads could be found at sites affected by periodontitis and peri-implantitis [4,8].

The detected concentrations of the tested periodontopathogenic marker bacteria tested with iai PadoTest^®^ (*F. alocis*, *P. gingivalis*, *P. intermedia*, *T. forsythia*, and *T. denticola*) were comparable between both sampling methods. In general, *A. actinomycetemcomitans* was rarely identified. This result is corroborated by another study that showed that the species were equally detected by PP and IDB [18]. Lower Ct values were detected for *T. denticola* around the teeth (healthy/gingivitis or periodontitis) and for *T. forsythia* around the teeth (healthy/gingivitis or periodontitis) and healthy implants (healthy/mucositis) using IDB compared to PP. Therefore, IDB may offer an advantage in their detection. This advantage could be due to the greater amount of DNA harvested by IDB in healthy conditions or gingivitis and mucositis. The possibility that mainly interdental biofilm is harvested may be less decisive, as it has many similarities with subgingival biofilm [25]. In addition, it can be assumed that a significant proportion of bristles is directed apically by the coronal pressure of the contact point, and thus a potentially decisive amount of subgingival plaque is also harvested. Therefore, IDB might have a particular value in the early (i.e., no clinically detectable periodontitis or peri-implantitis) detection of dysbiotic conditions. Furthermore, a rather superficial sampling, as with IDB, could explain the better detection of late colonizers, i.e., red complex (*T. denticola* and *T. forsythia*). This observation was also made by Belibasakis et al. regarding PP since curettes are more likely to remove biofilm at the tooth surface, while PP samples are more distant [11,14].

The frequent detection of all red complex bacteria, *F. alocis* and *P. intermedia*, in both healthy and diseased sites fit with the current literature [7,21]. *A. actinomycetemcomitans*, on the other hand, is rather rare [21]. In a comparison between healthy (and gingivitis/mucositis) and diseased sites, periodontopathogens can be found more abundantly in diseased sites, as previously shown [4,8].

The MDI values did not show significant differences between the two sampling techniques. In general, the values tended to be higher in diseased sites (i.e., periodontitis/peri-implantitis) when compared to healthier sites. In diseased sites, the samples from PP showed a tendency for higher MDI values and detected a higher number of PadoTest^®^ types three and five (indicative of more severe periodontitis). This might be explained by the fact that IDB is limited with the collection of biofilms located more subgingivally. In contrast, the samples from IDB detected more PadoTest^®^ types three and five as compared to the samples from paper points in healthy (and gingivitis/mucositis) patients. Moreover, the MDI with IDB tended to be elevated in this group. Therefore, IDB might be advantageous to sample plaque and detect a possible dysbiotic shift already at an earlier stage. This might be again due to the capability of IDB to harvest bigger amounts of plaque but also due to the fact they were used to probe interdentally where more plaque can be found. From a clinical point of view, this finding is of particular interest, as it can allow for a simple routine collection of biofilms during oral hygiene instructions while having a potential advantage in early diagnostics. Anyway, well-controlled longitudinal clinical studies will be needed to confirm this. A study by Santigli et al. [26], however, has already indirectly pinpointed that such a strategy could be especially valuable for healthy children for oral biofilm sampling in a standardized noninvasive procedure at an early stage. Thereby, they concluded that sampling with standardized PP, despite being an established and reliable method, remains challenging since the sampling with thin PP is limited in a sulcus of clinically (still) healthy gums.

Critically, it should be emphasized that this study did not differentiate between health and gingivitis or mucositis. However, this pilot study served as an initial evaluation of IDB for microbial sampling. Therefore, only a small number of participants were included in this study. It also seemed reasonable to initially differentiate solely between a clearly diseased (i.e., periodontitis/peri-implantitis) and a healthy or still reversibly inflamed situation. This aspect prevents a differentiated statement between health and gingivitis/mucositis. However, it also follows that a difference between healthy only and periodontitis/peri-implantitis would likely be greater than detected in the present study. Furthermore, paper points were always used to sample buccal and lingual surfaces while interdental brushes subsequently harvest biofilm of approximal surfaces. The intention of this approach was to prevent material from being translocated into the removal area of the interdental brushes via the paper points that were applied first. For this reason, when selecting the teeth, care was taken to ensure that the diseased areas had facially similarly increased probing depths as interdentally. In terms of probing depth, deep sites are generally advantageous in microbial sampling, while facial but also interdental surfaces are sampled in studies [13,25].

To summarize—despite the obvious shortcomings of a pilot trial—the results showed that a sampling method with interdental brushes was able to detect the marker pathogens used for iai PadoTest^®^ comparable to the collection with paper points (*F. alocis*, *P. gingivalis*, *P. intermedia*, *T. forsythia*, *T. denticola,* and *A. actinomycetemcomitans*). Higher concentrations of *T. denticola* and *T. forsythia* and a tendency for higher microbial dysbiosis index values, especially in healthy sites, were detected with interdental brushes in comparison to paper points. Therefore, interdental brushes might be a valid technique for microbial sampling and particularly advantageous in the early detection of dysbiotic shifts around teeth and implants. The use of interdental brushes could lead to a further reduction of invasiveness and might even be performed by the patient at home, as it is a routine oral hygiene procedure.

## Data Availability

All data are available on request.
